# Light Effects on Behavioural Performance Depend on the Individual State of Vigilance

**DOI:** 10.1371/journal.pone.0164945

**Published:** 2016-11-07

**Authors:** Ángel Correa, Antonio Barba, Francisca Padilla

**Affiliations:** 1 Centro de Investigación Mente, Cerebro y Comportamiento, Universidad de Granada, Granada, Spain; 2 Departamento de Psicología Experimental. Universidad de Granada, Granada, Spain; University of Pennsylvania, UNITED STATES

## Abstract

Research has shown that exposure to bright white light or blue-enriched light enhances alertness, but this effect is not consistently observed in tasks demanding high-level cognition (e.g., Sustained Attention to Response Task—SART, which measures inhibitory control). Individual differences in sensitivity to light effects might be mediated by variations in the basal level of arousal. We tested this hypothesis by measuring the participants’ behavioural state of vigilance before light exposure, through the Psychomotor Vigilance Task. Then we compared the effects of a blue-enriched vs. dim light at nighttime on the performance of the auditory SART, by controlling for individual differences in basal arousal. The results replicated the alerting effects of blue-enriched light, as indexed by lower values of both proximal temperature and distal-proximal gradient. The main finding was that lighting effects on SART performance were highly variable across individuals and depended on their prior state of vigilance. Specifically, participants with higher levels of basal vigilance before light exposure benefited most from blue-enriched lighting, responding faster in the SART. These results highlight the importance of considering basal vigilance to define the boundary conditions of light effects on cognitive performance. Our study adds to current research delineating the complex and reciprocal interactions between lighting effects, arousal, cognitive task demands and behavioural performance.

## Introduction

The current research addressed the effects of light on neurocognitive functions during task performance. This interest comes from evidence supporting that exposure to either bright white light or blue-enriched light enhances arousal and neural processes [1–3]. However, these alerting effects not always transfer to behavioural benefits. In fact, some studies have reported that performance can be either unaffected or impaired by light [4–9].

The heterogeneity between methodologies from different studies invites further research. In particular, physical factors of light determine its effects on performance, such as intensity, spectral composition, timing and duration of exposure [10–12]. Individual differences and psychological factors have been further considered to explain the variability of the cognitive effects of light. For example, light sensitivity depends on individual differences in the clock gene PER3 involved in sleep-wake regulation [13], age [14], cognitive domain [4,15] and task difficulty [16].

The Yerkes-Dodson Law [17] has been proposed to explain why light exposure may affect differentially to different cognitive tasks. This law describes the relationship between task performance and physiological arousal, which is a function of task difficulty. For easy, well-learned tasks, arousal increments would benefit performance linearly. For difficult tasks, this function would follow an inverted U shape, with poor performance at very low or high arousal level, and optimal performance at intermediate levels of arousal. Therefore, the alerting effect of light would influence performance depending on both task difficulty and the arousal level of individuals. However, individual’s arousal at the moment of light exposure had been rarely considered in lighting research.

The scarce research on this topic suggests a reciprocal interaction between light and arousal. While it is clear that light increases arousal, a few studies further suggest that variations in arousal can determine the extent to which light is effective. In particular, the presentation of a novel stimulus presumed to increase arousal in hamsters (a new running wheel), attenuated the synchronizing effect of light on circadian rhythms [18]. Moreover, extremely low basal arousal induced by sleep deprivation can block the alerting effect of light upon the activity of suprachiasmatic nuclei, but this effect can be restored by increasing arousal through administration of caffeine [19,20, 21].

These findings altogether highlight the importance of measuring the level of arousal in order to understand the differential sensitivity to light. The current research addressed the role of individual differences in the basal state of vigilance in relation to the behavioural effects of light. The individual state of vigilance before light exposure was measured behaviourally through the Psychomotor Vigilance Task (PVT) [22]. We then compared the effects of a blue-enriched vs. dim light on the performance of the Sustained Attention to Response Task (SART) [23].

The SART is a response inhibition task that requires to respond quickly to different single digits ranging randomly from 1 to 9 (go condition), except for the ‘3’ (no-go condition) to which participants must not respond. In a previous study [24], we found that inhibitory control was enhanced when participants performed the SART at their optimal time of day according to their chronotype (i.e., morning type participants performing in the morning, and evening-type in the evening). Since it is not always possible to schedule tasks at our optimal timing, the alerting effects of light could be an effective countermeasure to work at adverse circadian phases. Therefore, in the current experiment we tested whether nocturnal exposure to blue-enriched light would mimic the benefit of performing this task at optimal times of day. That is, we tested whether light can enhance the ability for response inhibition in the SART.

Distal and proximal skin temperatures were recorded to obtain a physiological marker of the alerting effects of light. Facilitation of somnolence and sleep at night has been related to increments in the distal-proximal temperature gradient (DPG) [25], whereas exposure to blue light at night decreases the DPG suggesting alertness enhancement by triggering the sympathetic tone [2].

We expected that blue-enriched white light, in relation to the dim light condition, would enhance alertness as indexed by lower DPG and faster responses in the PVT. We further expected that the basal level of behavioural arousal (measured by the PVT before light exposure) would mediate the behavioural effects of blue-enriched light on response inhibition during the SART.

## Materials and Methods

### Participants

Twenty-two psychology students (15 females, *M =* 19.91; *SD* = 3.24) from the University of Granada participated voluntarily in the experiment. The inclusion criteria were having slept a minimum of 6 hours previous to the experimental session, no intake of psychoactive substances and having intermediate chronotype. Four participants slept less than 6 hours and three participants did not come to the second session. The final sample finally included 15 subjects. The study was approved by the Ethics Committee of the University of Granada (n.34/CEIH/2015). All participants gave prior written informed consent and they were rewarded with course credits at the end of the experiment.

### Apparatus and Stimulus

#### Lighting

**Fig 1:**
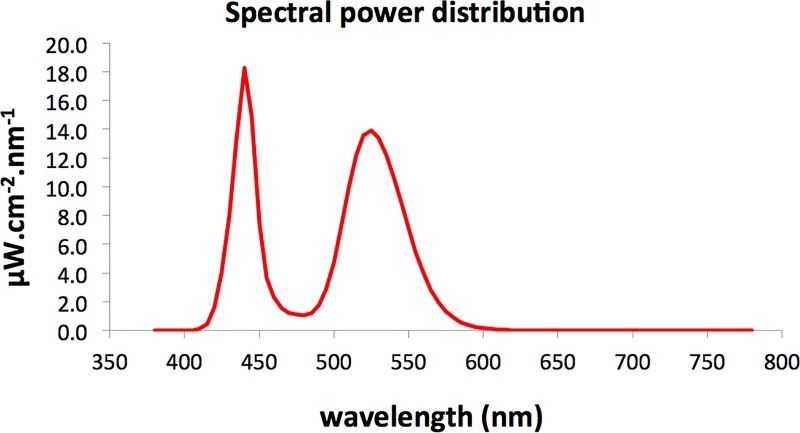
Spectral power distribution with melanopic weighted power of the blue-enriched light.

A matrix of 16 light emitting diodes—LEDs (*IgniaLight*, *SACOPA*, *S*.*A*.*U*.*)* emitted blue-enriched light with a peak about 440 nm. The spectral power distribution was measured at the eye level with a calibrated spectroradiometer (Ocean Optics Ltd) and is displayed in Fig 1.

Illuminance measurements for each of the retinal photoreceptors, calculated by the toolbox developed by Lucas et al. [26], are presented in Table 1.

**Table 1:** Equivalent α-opic illuminance values for the blue-enriched light condition.

	λ_max_	α-opic lux
Melanopsin	480.0	1805.7
S-cone	419.0	2566.4
M-cone	530.8	3246.7
L-cone	558.4	3580.6
Rods	496.3	2384.0

#### Questionnaires and Scales

The Morningness–Eveningness Questionnaire (MEQ) [27] measured the participant’s chronotype. Scores can range from 16 to 86, including evening (16–41), intermediate (42–58) and morning chronotypes (59–86). We used a general interview about health and sleep habits including questions on the day and night previous to the experiment (the full version of the health and sleep habits questionnaire can be found in S1 File). The Karolinska Sleepiness Scale (KSS) [28] measured the subjective level of sleepiness in our participants before and after light exposure, ranging from 1 (“totally alert”) to 9 (“maximum sleepiness”). Likewise, in a 9-point likert mood scale (further details in S1 File), participants scored between 1 (“extremely negative”) and 9 (“extremely positive”). Finally, a 10-likert scale asked about the perceived mental effort to perform the main cognitive task (SART) and the level of visual comfort under lighting regarding three items (agreeableness, intensity of light and preference of the blue-enriched light to work/read”. All questionnaires and scales were computerized and presented visually over a black background.

#### Physiological recording

Samples of body temperature were recorded every minute using a sensor (*iButton—DS1921H; Maxim*, *Dallas*), with a temperature range between +15°C and +46°C and 1°C of accuracy with a precision of 0.125°C. Sensors were placed with adhesive tape in every participant as follows: in the non-dominant wrist to record distal skin temperature, in the left infraclavicular area to record proximal skin temperature, and in the nose as additional measure of proximal skin temperature. Room temperature was also registered at the start and the end of the experiment using a clock-thermometer (*Inovalley–F95310*).

#### Behavioural Tasks

All tasks were delivered through E-prime 2.0 [29] and presented on a 17-inch computer screen connected to an Intel Core 2 computer. Headphones (*Seinheiser HD 201*) were used in auditory tasks. Auditory stimuli were presented at a comfortable but clearly audible intensity (60% of the maximum level), which was constant to all participants.

*Auditory Sustained Attention to Response Task (SART)*. The SART is a *go/no-go* task [23], which was adapted to the auditory domain [30] to measure non visual effects of light on cognition. Participants had to respond as quickly as possible to a random sequence of simple auditory digits ranging between 1 and 9 (*go* trials), except for the digit “6”, to which participants have to inhibit response (*no-go* trials). We used “6” as no-go stimulus to increase task demands, since the first phoneme (/S/) is similar to other ‘go’ stimuli in Spanish (e.g.: “5”, “7”). The auditory digits were presented randomly in one of five different tone frequencies (two high pitch, one intermediate and two low pitch), trying to simulate the original SART, in which perceptual variability relied on visual digits displayed in different font sizes. A list of 240 trials was presented in fully randomized order, from which 200 were *go* trials (25 trials per each go digit) and 40 were *no-go* trials. Each trial consisted of a black screen during 50 ms, followed by the target auditory stimulus until either participant’s response or 1150 ms. The SART lasted 40 minutes without any breaks to study time on task effects on vigilance [24].

*Auditory Psychomotor Vigilance Task (PVT)*. Participants were to respond as quickly as possible to the onset of a target tone (700 Hz, 250-ms duration) appearing after a black screen which duration varied randomly on every trial between 2000 and 10000 milliseconds. Feedback (for 1000 ms) on participant’s performance (reaction time–RT- and anticipations) was delivered visually. The PVT lasted 10 minutes.

### Procedure

Before the experiment, participants completed a sleep habits interview and the reduced version of the MEQ (rMEQ) [31]. The selected participants completed the study in two night sessions separated by at least 7 days, both at 10 pm, only differing in the lighting condition (blue-enriched light vs. dim light). Every participant was tested in both blue-enriched light and dim light conditions, which order was counterbalanced across participants. When participants arrived at the laboratory, temperature sensors were placed on distal (non-dominant wrist) and proximal (left infraclavicular and nose) areas.

Then, they performed the PVT, KSS and mood scales under dim light. After this, participants started a 15-minute period of adaptation to the lighting condition. Subjects were seated in front of the LEDs lamp with their head on a chin rest to hold a fixed distant between eyes and light source. In both lighting conditions the same lamp was turn on, but it was fully covered by an opaque box during the dim light condition (≤3 lux). Subsequently, they completed the general interview and MEQ through the computer. They performed the auditory SART wearing the headphones. We encouraged participants to maintain their eyes gaze in a fixation point located on the wall throughout the task. Once the SART was finished we turned off the LEDs lamp and each participant performed again the PVT, KSS and mood scales, and completed the mental effort and visual comfort scales. The experimenter recorded room temperature at the beginning and end of sessions.

### Design and data analysis

Potential differences between the two lighting sessions were considered by separate repeated-measures analyses of variance (ANOVA) with the factor of Lighting (blue-enriched light and dim light) on the following dependent variables: chronotype (MEQ score), sleep duration and time awake before experiment (in hours), room temperature variation (final minus initial measurements), and perceived mental effort.

Since the KSS, mood scale, and PVT were administered twice, Testing time (pre-lighting and post-lighting) was added as within-subject factor. The analysis of mean inverse RTs in the PVT did not include trials with RTs below 100 ms (anticipations).

Skin temperature was computed through wrist, infraclavicular and nasal temperatures during the SART, corrected by a baseline during the period of adaptation to lighting (i.e., an average of 10 minutes recording just before the SART). The distal to proximal temperature gradient (DPG) was computed as usual, by subtracting distal (wrist) minus proximal (infraclavicular) temperatures. Time on task effects were analyzed by including Blocks of 10 minutes as a factor with four levels.

The RT analysis in the SART included correct responses in go-trials with RTs within 2.5 SD from the mean (2.32% excluded by subject, session and condition). The accuracy analysis computed the proportion of correctly inhibited responses in no-go trials. Mean RT and accuracy were submitted to separate analyses of covariance (ANCOVA) with the factors of Lighting (blue-enriched light and dim light) and Block (1, 2, 3 and 4), controlling for Basal Vigilance. Each participant’s basal vigilance was computed as the (inverse) RT difference between pre-lighting sessions in the PVT administered before light exposure. Therefore, positive scores in basal vigilance meant faster PVT performance in the blue-enriched vs. dim light sessions just before the lighting manipulation.

All analyses including significant effects of Block were further analyzed by linear trend analysis. The Greenhouse-Geisser correction was applied, and corrected probability values and degrees of freedom were reported, when sphericity was violated [32]. Data are available in S1 Data.

## Results

### Demographic Data

The ANOVA showed no differences between blue-enriched and dim light conditions in either the MEQ scores, sleep duration, time awake before the experiment or ambient temperature variation (all *Fs <* 1). Descriptive statistics are presented in Table 2.

**Table 2:** Scores (mean and standard deviation) in demographic variables for blue-enriched and dim light conditions.

	*Blue-enriched Light*		*Dim Light*	
	*M*	*SD*	*M*	*SD*
MEQ scores	50.13	5.78	50.13	5.19
Sleep duration (hours)	7.63	0.75	7.79	0.67
Time awake before the experiment (hours)	13.21	0.82	12.94	0.72
Room temperature variation (in °C)	1.55	0.40	1.61	0.29

### Body temperature

**Fig 2:**
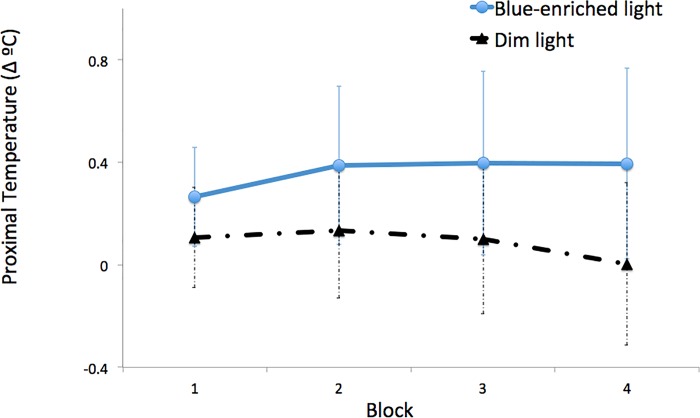
Variation in infraclavicular temperature with respect to baseline as a function of time on task (block) and lighting (blue thick line: blue-enriched light, black thin line: dim light).

The Lighting (blue-enriched light and dim light) x Block (1, 2, 3 and 4) analysis showed higher infraclavicular temperature for blue-enriched light vs. dim light conditions (*M =* .36; *SD* = .09 and *M* = .09; *SD* = .08; *F*(1, 14) = 5.92, *p* = .03, *η*_*p*_^*2*^ = .30). The interaction between *lighting* and *block*, *F*(1.79, 25.10) = 5.67, *p* = .01, *η*_*p*_^*2*^ = .29, indicated that temperature evolved differentially along time on task as a function of lighting. Further analyses revealed a marginal linear decrement in dim light, *F*(1, 14) = 3.54, *p* = .08, but not in the blue-enriched light condition, *F*(1, 14) = 2.67, *p* = .12 (Fig 2).

The distal-proximal gradient was lower in blue-enriched light vs. dim light conditions (*M = -*.43; *SD* = .17 and *M* = .06; *SD* = .22; *F*(1, 14) = 6.12, *p* = .03, *η*_*p*_^*2*^ = .30). Wrist and nasal temperatures showed no significant effects (all *p*s > .13)

### Subjective measures

Lighting (blue-enriched light and dim light) x Testing time (pre-lighting and post-lighting) ANOVAs showed higher subjective sleepiness after (vs. before) the SART, *F*(1, 14) = 61.05, *p* < .001, *η*_*p*_^*2*^ = .81 (M = 7.10, SD = .19 and M = 4.60, SD = .35, respectively). Likewise, subjective mood became less positive after the SART, *F*(1, 14) = 10.80, *p* = .005, *η*_*p*_^*2*^ = .44 (M = 6.93, SD = .22 and M = 6, SD = .40). In the mental effort scale, the main effect of *lighting* was not significant, *F*(1, 14) = 2.73, *p* = .12. In the comfort scale, subjects considered the blue-enriched light intermediate in terms of agreeableness (M = 4.6), and preference *to work/read* (M = 4.4), and relatively high regarding intensity (M = 6.27).

### Auditory Psychomotor Vigilance Task (PVT)

The Lighting (blue-enriched light and dim light) x Testing time (pre-lighting and post-lighting) ANOVA on the mean inverse RTs showed a significant effect of *lighting*, *F*(1, 14) = 5.08, *p* = .04, *η*_*p*_^*2*^ = .27, with faster responses in the blue-enriched light than in dim light. Responses were also faster before than after the SART, Testing time: *F*(1, 14) = 68.64, *p* < .001, *η*_*p*_^*2*^ = .83. However, the interaction between lighting and testing time was not significant (*F <* 1). That is, the vigilance decrement along the experiment (i.e., slower responses after the SART) was not prevented by lighting. In fact, a specific comparison in the pre-lighting condition suggested faster responses in the blue-enriched light session than in the dim light session, *F*(1, 14) = 3.55, *p* = .08. In other words, the basal vigilance state was already different between conditions before lighting exposure. Therefore, the following analyses considered this potentially confounding influence in the effects of lighting by including the participants’ basal vigilance as a covariate.

### Auditory Sustained Attention to Response Task (SART)

**Fig 3:**
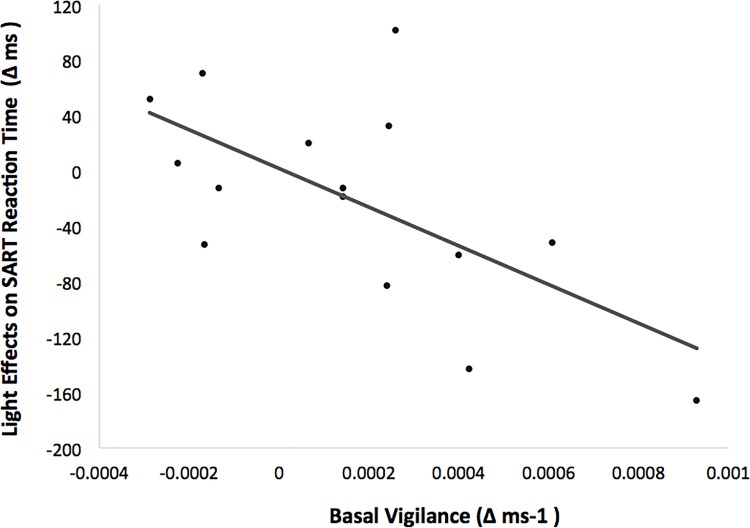
Correlation between basal vigilance (inverse reaction time difference between pre-lighting sessions in the Psychomotor Vigilance Task -PVT- administered before light exposure) and the effects of light on reaction times in the Sustained Attention to Response Task (SART).

The Lighting (blue-enriched light and dim light) x Block (1, 2, 3 and 4) ANCOVA controlling for Basal Vigilance performed on SART reaction times showed a main effect of *block*, *F*(1.60, 20.74) = 9.11, *p* = .003, *η*_*p*_^*2*^ = .41, revealing a linear decrement in RT with time on task, *F*(1, 13) = 14.22, *p* = .002. Most important, there was a significant effect of lighting after controlling for basal vigilance, *F*(1, 13) = 8.25, *p* = .013, *η*_*p*_^*2*^ = .39. This effect remained stable with time on task (lighting x block: *F <* 1). Fig 3 plots the covariation between basal vigilance and light effects on RT performance in the SART (negative values meant faster RTs in blue-enriched vs. dim light condition).

This figure shows that blue-enriched lighting produced larger improvements in response speed with higher levels of basal vigilance (linear correlation, *r* = -.62, *p =* .013). In other words, lighting effects on performance were variable across individuals depending on their prior behavioural state of vigilance.

A similar ANCOVA was performed on the accuracy to inhibit responses in the no-go condition. The analysis revealed a main effect of *block*, *F*(2.08, 26.98) = 17.50, *p* < .001, *η*_*p*_^*2*^ = .57, indicating lower accuracy with increasing time on task, *F*(1, 13) = 43.59, *p* < .001 (averages for block 1: .81 and block 4: .67). After controlling for basal vigilance, the effect of lighting was not significant and it did not interact with block (both *ps >* .13).

#### Behavioural vs. physiological basal vigilance

Basal vigilance could be measured not only behaviourally, but also at a physiological level by using the DPG. We therefore replicated the previous ANCOVAs, now by controlling for basal physiological arousal. Likewise, basal physiological arousal was computed as the DPG difference between sessions during the period just before light adaptation (i.e., in each lighting session, the DPG values, recorded while participants completed the subjective scales, were averaged together).

The Lighting (blue-enriched light and dim light) x Block (1, 2, 3 and 4) ANCOVAs controlling for basal physiological arousal performed on SART reaction times and accuracy showed no significant main effects or interactions involving the lighting factor (all *ps >* .36). Note that data from two subjects could not be included in this DPG analysis, as their distal temperatures had not yet reach normal physiological values (< 29°C). Therefore, similar ANCOVAs were further performed, using infraclavicular temperature instead of DPG to estimate basal physiological arousal with the full sample. However, this analysis did not show either significant main effects or interactions involving the lighting factor (all *ps >* .48).

This dissociation between behavioural and physiological measures of basal arousal was further tested by a linear correlation between both covariates (inverse RTs in the pre-lighting PVT and the DPG), which was not significant (*r =* .28, *p =* .31). Also in contrast to the results from behavioural measures, t-tests conducted on physiological measures (DPG and proximal temperature) of basal vigilance revealed that participants’ physiological states before lighting did not differ between the two sessions (p = .42 and p = .19).

## Discussion

The effect of lighting on physiological measures and behavioural performance in simple tasks (PVT) had been previously reported [11,33–35]. However, this behavioural effect remains elusive when complex tasks demanding cognitive control are used [8,9,15]. Recent studies [4,16] suggest that light effects may be mediated by the cognitive demands of the task (i.e., difficulty). According to the Yerkes-Dodson law [17], arousal increments due to light exposure would exert differential effects on performance depending on cognitive demands. However, participants’ basal level of arousal had not been considered by previous research, to the best of our knowledge.

The current study addressed, for the first time, the role of individual differences in basal vigilance state on the behavioural effects of light, during the performance of an inhibitory control task. Participants completed auditory versions of the PVT and SART under blue-enriched and dim light conditions in the early night (10 pm). We expected that blue-enriched light enhanced vigilance as indexed by skin temperature and the PVT, and that this increment would also be reflected in SART performance. Two main findings emerged. First, we dissociated between physiological and behavioural effects of light: blue-enriched light enhanced vigilance as indexed by skin temperature, whereas this physiological effect was not evident at the behavioural level as indexed by the PVT [36]. Second, we found that behavioural effects of light on SART performance were highly variable across individuals, and depended on their prior state of vigilance.

Analyses of skin temperature in the dim light, control condition, replicated the typical decrement in proximal temperature at nighttime, suggesting higher somnolence over time [2]. In contrast, blue-enriched light exposure effectively attenuated such a decrement, suggesting an alerting effect of light. Furthermore, the finding of lower DPG under blue-enriched lighting supported a reduction of participants’ somnolence at nighttime, presumably by preventing vasodilation through the engagement of the sympathetic nervous system. High values of DPG involve a reduction of core body temperature by dissipation of heat via vasodilation of distal areas, which facilitates sleep onset [25]. However, nasal temperature was not sensitive to vigilance changes in our experiment. This measure was proposed to index subjective workload, mental stress or negative emotions associated with the Autonomic Nervous System [37,38].

In line with other studies [4,7,36], the results from the PVT did not corroborate a clear alerting effect of light on behaviour. However, considering this behavioural measure was crucial to our main finding. That is, the participant’s vigilance state before light exposure can modulate the subsequent behavioural effects of light. We specifically found that participants with higher levels of basal vigilance benefited most from exposure to blue-enriched light, as revealed by faster responses in the SART. This finding makes sense in the context of research suggesting that arousal mediates acute effects of light. Indeed, studies reporting differential sensitivity to light exposure probably involved manipulations in basal arousal, for example, due to presentation of novel stimuli, sleep deprivation, adverse circadian phase, mental fatigue, caffeine intake or demanding tasks [4,13,15,16,18–21,39].

The current findings further highlighted a dissociation between behavioural (PVT performance) and physiological (DPG) markers of basal arousal. Only basal behavioural vigilance, but not basal physiological arousal, differed across lighting conditions and covaried with the behavioural effects of light. Linear correlation analyses did not support either a relationship between behavioural and physiological levels of basal vigilance. Basal differences in psychological states (e.g., related to motivation) that were captured behaviourally by the PVT (but not physiologically by temperature), might be a natural consequence of studying humans in free-living conditions. Hence, a muldimensional consideration of basal vigilance (including subjective, behavioural and physiological variables) may help clarify divergent results concerning the effects of light on behavioural performance.

As already suggested [4,7,16,21], the Yerkes-Dodson law [17] would explain how the arousing effects of light could lead to different results depending on the initial state of the individual. For example, under very low levels of basal arousal, light could be ineffective, but this effect can be restored by caffeine administration [19]. In agreement with the latter study, our results revealed a positive relationship between arousal and lighting effects. In contrast, other studies have suggested that light could be most effective in adverse conditions, such as performing at non-optimal times of day and after 1-night of sleep deprivation [13,39] or under mental fatigue [7]. Further research is hence granted as behavioural measures still show mixed results in different cognitive tasks.

At the neural level, interactions between arousal and light effects might be mediated by the connection between the intrinsically photosensitive retinal ganglion cells (ipRGC) and subcortical structures involved in the regulation of arousal, circadian rhythms and sleep, such as the paraventricular thalamic nucleus [39–41]. Innervations of the paraventricular thalamic nucleus to the prefrontal cortex, nucleus accumbens and amygdala containing orexin could modulate the cortical activation underlying the behavioural effects of light.

Regarding task difficulty in the Yerkes-Dodson law, it is interesting to note that our accuracy analysis (response inhibition to no-go trials) did not reveal any lighting effects. Thus, the RT benefit induced by light in highly vigilant participants was not achieved at the cost of impaired response inhibition in this experiment. It has been proposed [23] that the SART can measure both automatic and controlled response modes. Reaction times in the go condition reflect the automatic mode of participants’ response, given the repetitive and frequent demand of quick responses. Accuracy in the no-go condition reflects controlled processing, as participants have to perform inhibitory control over the automatic tendency to respond. Our findings supported this dissociation by showing selective effects of light in the automatic but not in the controlled process. Since controlled response inhibition is more demanding than automatic responding, and according to the Yerkes-Dodson law, we assume that light manipulation increased arousal, leading to faster RTs in the “easy” (go) condition, but not in the accuracy measure of our “difficult” (no go) condition. Indeed, the increment in response speed with arousal followed a linear trend, as predicted by the Yerkes-Dodson law for easy tasks. As abovementioned, other studies have shown that behavioural effects of light depend on the cognitive demands of the task at hand [4,15], and are not easily observed for challenging, complex tasks [12].

## Conclusions

The current research replicated that blue-enriched light enhances alertness during early night. Moreover, we show for the first time that individual differences in basal vigilance state can determine how light affects cognitive performance. This result is relevant to understand why research on the effects of light on cognition has provided divergent findings. Thus, the stimulating effects of light can exert positive, null or negative effects on task performance depending on the individual’s neurobehavioural status. Therefore, a mixture of opposite effects, through statistical group averaging without considering basal vigilance, could mask interesting effects of light on behaviour. Future research should manipulate basal vigilance intra-individually to gain understanding on the complex interactions between light effects, arousal, cognitive demands and performance.

## Supporting Information

S1 File Questionnaire on health and sleep habits, Mood 9-point Likert Scale. (PDF)

S1 Data Averaged data per subject and experimental condition. (XLS)

## Abbreviations

RT: reaction time
PVT: psychomotor vigilance task
SART: sustained attention to response task
DPG: distal-proximal temperature gradient
M: mean
SD: standard deviation
LED: light emitting diode
MEQ: morningness-eveningness questionnaire
KSS: Karolinska sleepiness scale
ANOVA: analysis of variance
ANCOVA: analysis of covariance
ipRGC: intrinsically photosensitive retinal ganglion cells.
