# Supplementary material for: Light Effects on Behavioural Performance Depend on the Individual State of Vigilance
Source: PLoS One. 2016 Nov 7;11(11):e0164945. doi: 10.1371/journal.pone.0164945 (PMC5098788; doi:10.1371/journal.pone.0164945)
Supplement: S1 File — (PDF) [file pone.0164945.s001.pdf]

Supporting Information

Questionnaire on health and sleep habits

- What time did you go to bed last night?
- What time do you think you fell asleep?
- How many hours do you think you slept last night in total?
- Have you had a nap? (indicate when)
- Have you had any stimulants (tea, coffee, caffeine drinks...)? How many and when?
- Have had any relaxing substances (lime blossom, b-blockers, ansiolitics) before sleeping?
- Have you had any alcoholic drink today?
- Have you had any alcoholic drink last night?
- Have you had any drug last night?
- How many hours have you spent working/studying today (indicate when)?
- Have made physical exercise today? (If so, indicate, activity and when)

Questionnaire on health and sleep habits (Spanish)

-¿A qué hora te acostaste anoche?
-¿A qué hora crees que te dormiste?
-¿A qué hora te has despertado?
-¿Cuántas horas crees que dormiste anoche en total?
-¿Has dormido siesta? ¿De qué hora a qué hora?
-¿Has tomado alguna sustancia estimulante (té, café, bebidas con cafeína…)?
¿Cuántas? ¿A qué hora tomaste la última?
-¿Has tomado alguna sustancia relajante (tila, b-bloqueantes, ansiolítico…)?
-¿Consumiste anoche alguna sustancia relajante (tila, b-bloqueantes, ansiolítico…) antes de ir a dormir?
-¿Has consumido hoy alguna bebida alcohólica?
-¿Consumiste anoche alguna bebida alcohólica?
-¿Tomaste algún medicamento anoche?
-¿Cuántas horas has pasado hoy trabajando y/o en clase (en qué horario)?
- ¿Has practicado hoy algún deporte o actividad física?
- Si has señalado el SÍ, contesta a las siguientes preguntas. Tienes espacio para responder con respecto a dos actividades físicas diferentes.
¿Qué actividad has practicado?
¿A qué hora la has practicado?

Mood 9-point Likert Scale

“Please, indicate what your current mood is by pressing a number between 1 and 9, considering 1 as a mood ‘extremely bad and 9 ‘extremely good”

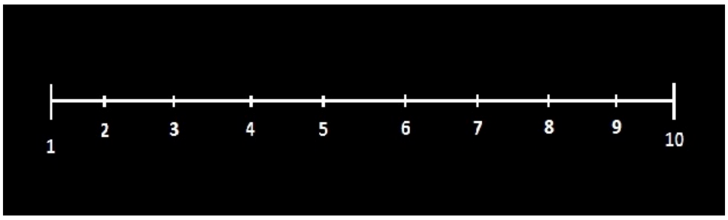

Mood 9-point Likert Scale (Spanish)

Por favor, indica cuál es tu estado de ánimo actual con un número del 1 al 9, considerando el 1 como un estado de ánimo "extremadamente malo" y el 9 como un estado de ánimo "extremadamente bueno".
